# Drug Errors in Anaesthesiology

**Published:** 2009-10

**Authors:** Rajnish Kumar Jain, Sarika Katiyar

**Affiliations:** 1 Professor and Head, Department of Anaesthesiology and Critical Care, Bhopal Memorial Hospital and Research Centre, Bhopal-462038, India

**Keywords:** Drug errors, Anaesthesia, Medication Errors

## Abstract

**Summary:**

Medication errors are a leading cause of morbidity and mortality in hospitalized patients. The incidence of these drug errors during anaesthesia is not certain. They impose a considerable financial burden to health care systems apart from the patient losses. Common causes of these errors and their prevention is discussed.

## Introduction

Medication error is a leading cause of morbidity and mortality in hospitalised patients. The practice of anaesthesiology requires the administration of a wide variety of potent medications. These medications are often given in high acuity situations and in environment with poor visibility and multiple distractions. Medications with widely differing actions such as muscle relaxants, vasopressors and vasodilators are often used in the course of a single anaesthetic, at times simultaneously. Due to high potency, variety and frequency of drugs administered to patients undergoing anaesthesia, the potential exists for errors with disastrous consequences.

The Joint Commission in USA has made reduction of medical errors one of its National patient safety goals for the past several years. In 2006, the FDA mandated that manufacturers include a machine readable bar code, radiofrequency identification (RFID), and computerised ordered entry (CPOE) systems have all come on the horizons as technological solutions, only to create different problems which may be almost as big as those they are intended to solve.^1^

It is painfully obvious; we are yet nowhere near a solution, even for so called “high alert medication”. The administration of flush solution with a heparin concentration 1000 times that intended to seventeen Texas neonates in Corpus Christi on July 4^th^ last year is just one very recent example of how far we have to go 2.

Drug errors represented 4% claims in the ASA Closed Claims Project Report (2003). For ease, drug errors may be classified into the following categories (after Webster etal) 3.

1. Omission–Drug not given
2. Repetition-Extra dose of an intended drug
3. Substitution-Incorrect drug instead of the desired drug: a swap
4. Insertion-A drug that was not intended to be given at a particular time or at any time
5. Incorrect dose-Wrong dose of an intended drug
6. Incorrect route-Wrong route of an intended drug
7. Others-Usually a more complex event, not fitting the above categories

**Fig 1:**
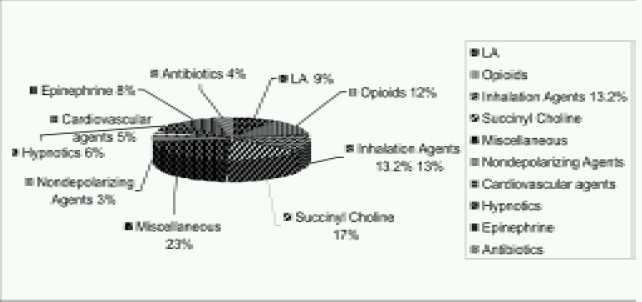
Types of drugs involved in drug errors

As per reports of Closed Claims Project (2003), out of 205 claims for drug errors, there were only two cases of “omission”, four cases of “incorrect route”, and no cases of “repetition”. There were 50 cases of “substitution” (24%), 35 cases of “insertion” (17%), 64 cases of “incorrect dose” (31%), and 50 cases of “others” (24%). Drug infusions were involved in 30 cases (15%). Most common drug involved in infusion was succinyl choline. Drug administration errors frequently resulted in serious problems. There were 50 deaths (24%) and 70 cases with major morbidity. A wide variety of drugs were involved in errors (Fig 1). Two drugs in particular were most commonly involved. Succinyl choline was involved in 35 cases (17%) and Epinephrine was involved in 17 cases (8%).^4^

Medical literature is replete with anecdotal reports of medication errors that raise disturbing questions. How often do such tragedies occur in India? Unfortunately we don't know the answers to these questions because no mechanism exists to track medication errors or to develop strategies to prevent their recurrence. Significant research has already been done to evaluate drug administration procedures and technology to improve safety of drug administration during anaesthesia. These techniques are summarised in Table-1^1^,^5^–^14^

**Table 1:** Error Reduction technique

1. Pre-filled syringes
2. Distinctive Drug Labels
3. Coloured Drug Labels
4. Check labels with second observer
5. Double Check Ampoule before labelling syringe and syringe label before administration
6. Do not store concentrated solutions o f hazardous medications(KCl) in OT
7. Standardization of drug preparation procedures
8. Standardization of layout of drug workspace
9. Standardization –syringe sizes
10. Bar codes on drug labels with audible reader

However, major cause of drug error is misidentification of drug ampoules or vials. Confusing, inaccurate or incomplete labels contributed to 21% of the actual or potential drug errors reported to the US Pharmacopoeia practitioners network over a one year period (1999).^14^ The recognition and identification of an object depends on the shape, colour, brightness and contrast. As these elements become increasingly distinctive, identification of the object becomes faster and more accurate.^15^–^17^ Therefore, although multiple factors contribute to medication errors, consistency and clarity of pharmaceuticals and syringe labelling in accordance with human factors are important elements in their prevention.

American Society of Anaesthesiologists supports the manufacture and use of pharmaceuticals with labels meeting the following standards which are consistent with those established by American Society for Testing and Material (ASTM) International. The main change to the drug label is the introduction of a critical information panel or field. The label presents the generic name of the drug, the total amount per total volume and the drug concentration in black text on a white background. In addition, the drugs proprietary name, manufacturer, lot number, date of manufacture and expiry date should also be included on the label. The text on the label should be designed to enhance the recognition of the drug name and concentration as recommended in the ASTM international standards.^18^

Maximum Contrast between the text and the background should be provided by high contrast colour combinations as specified in ASTM international Standard (Table- 2), which also minimise the impact of colour blindness.^18^

**Table 2:** Contrasting Background for labels

Text	Background
Black	White
Blue	Yellow
White	Blue
Blue	White

Nine Classes of drugs commonly used in practice of anaesthesiology have a standard background colour established for user applied syringe labels by ASTM international standards. For these drugs the colour of the containers top, label border, and any other coloured area on the label, excluding the background as required for maximum contrast, should be the colour responding to the drugs classification (Table- 3).^18^ Essential information including the drugs generic name, concentration and volume of the vial or ampoule should be bar coded at a location on the vial or ampoule which will not interfere with the labels legibility as specified in ASTM international Standard.^17^

**Table 3:** Drug Class and pantone colours

	Drug Class	Pantone Colour
1.	Induction Agents	Yellow
2.	Tranquilizers	Orange
3.	Muscle Relaxants	Florescent Red
4.	Relaxant Antagonists	Florescent Red/White diagonal Stripes
5.	Narcotics	Blue
6.	Narcotic Antagonists	Blue/White Diagonal Stripes
7.	Major Tranquilizers	Salmon
8.	Narcotic/Tranquilizer combination	Blue/Salmon
9.	Vasopressors	Violet
10.	Hypotensive Agents	Violet/White Diagonal Stripes
11.	Local Anaesthetics	Gray
12.	Anticholinergic Agents	Green

There are risks associated with using these colour coded syringes unless certain actions are taken to prevent syringe mix-ups that could prove harmful to patients. Colour coding for user applied labels was promoted for anaesthesia providers only in Operation Theatres. It was not designed for commercial product labels. Commercially packaged colour coded syringes also have different easily recognisable colours for various pharmacological classes of anaesthesia drugs. But a serious risk exists; there are often multiple drugs within a class, each with very different properties. These drugs are all available in the same colour and perhaps the same size syringes.^19^ Unlike anaesthesia providers who typically use a single drug within each class, commercial systems used to the fullest extent will result in many different agents within a class that share the same coloured syringe, risking drug selection errors. For example, it is possible to have three drugs, morphine, fentanyl and Pentazocine – each with significant potency variations, all in blue syringes in the same physical area. Mix-ups among these drugs could cause serious harm.

Colour coding strategies have led to repeated mixups ever since FDA allowed ophthalmologists and eye medication manufacturers to use a colour code classification system for classes of eye drop medications. This is problematic when staff other than ophthalmologist dispense or administer these drugs.^19^

While injuries may not be serious when a mix-up occurs between various eye drops, mix-ups between powerful anaesthesia drugs, mostly all high alert medications can prove fatal. Within the Operation Theatre (OT) most patients are intubated, monitored and have immediate care available in case of serious mix-ups. Outside the OT, mix-ups may be more difficult to recognise and manage quickly. To reduce the risk of these mix-ups commercial repackagers should label these products with warnings to encourage use by anaesthesia providers within the OT only. Standards should be modified by including drug names along the borders and additional colours along the edges to help differentiate products within each class. Bar coding systems would likely prevent most mix-ups; there is a barcode on these commercially available syringes.^19^

The next step towards a safer drug delivery system is the establishment of an agency for reporting medication errors. This agency would be similar to the American institute of safe medical practice (ISMP).^14^ The Med Marx programme of the US Pharmacopoeia (www.usp.org) enables health care professionals to report anonymously their concerns regarding the quality, safety, performance or design of products used in their practice .This non profit organisation identifies causal mechanisms and develop strategies to prevent their recurrence. This information is then shared with industry and appropriate government Agencies.

An investigation by Fasting et al showed that drug errors are uncommon and represent a small part of anaesthesia problems but still have the potential for serious morbidity.^13^ According to them syringe swaps occurred between syringes of equal sizes and were not eliminated by colour coding of labels.As per this study, system improvement by better visual cues and better checking procedures are needed.^13^ Reading the label before giving the drug is still the last in the line of multiple defences against the problem of drug error.

As evidenced by the latest JCAHO sentinenel events report (2009), anaesthesiology speciality continues to grapple with suboptimal outcomes, mostly related to poor communication.^20^ Anaesthesia providers work hours and practice settings are more variable than in the past and attendant breakdowns in clinical information transfer can lead to systemic medical errors. Current evidence indicates that improving clinical communication can also reduce medical error to some extent.

Thus, drug errors are an inevitable consequence of the human condition, they occur even among the most conscientious medical professionals. During the past two decades anaesthesiology has led many patient safety improvement initiatives and morbidity and mortality rates have decreased greatly as a result.
